# Implementation of the global plan for insecticide resistance management in malaria vectors: progress, challenges and the way forward

**DOI:** 10.1186/s12936-015-0693-4

**Published:** 2015-04-23

**Authors:** Abraham P Mnzava, Tessa B Knox, Emmanuel A Temu, Anna Trett, Christen Fornadel, Janet Hemingway, Melanie Renshaw

**Affiliations:** World Health Organization, Avenue Appia 20, 1211 Geneva, Switzerland; Swiss Tropical and Public Health Institute, Socinstrasse 57, 4051 Basel, Switzerland; University of Basel, Petersplatz 1, 4051 Basel, Switzerland; President’s Malaria Initiative, US Agency for International Development, 1300 Pennsylvania Avenue, 20523 Washington, DC USA; Liverpool School of Tropical Medicine, Pembroke Place, Liverpool, L3 5QA UK; Africa Leaders Malaria Alliance, Nairobi, Kenya

**Keywords:** Insecticide resistance, Malaria vectors, GPIRM, Resource deficiencies

## Abstract

In recent years, there has been an increase in resistance of malaria vectors to insecticides, particularly to pyrethroids which are widely used in insecticide-treated nets. The Global Plan for Insecticide Resistance Management in malaria vectors (GPIRM), released in May 2012, is a collective strategy for the malaria community to tackle this challenge. This review outlines progress made to date and the challenges experienced in the implementation of GPIRM, and outlines focus areas requiring urgent attention. Whilst there has been some advancement, uptake of GPIRM at the national level has generally been poor for various reasons, including limited availability of vector control tools with new mechanisms of action as well as critical financial, human and infrastructural resource deficiencies. There is an urgent need for a global response plan to address these deficits and ensure the correct and efficient use of available tools in order to maintain the effectiveness of current vector control efforts whilst novel vector control tools are under development. Emphasis must be placed on enhancing national capacities (such as human and infrastructural resources) to enable efficient monitoring and management of insecticide resistance, and to support availability and accessibility of appropriate new vector control products. Lack of action by the global community to address the threat of insecticide resistance is unacceptable and deprives affected communities of their basic right of universal access to effective malaria prevention. Aligning efforts and assigning the needed resources will ensure the optimal implementation of GPIRM with the ultimate goal of maintaining effective malaria vector control.

## Background

Between 2000 and 2013, worldwide malaria morbidity and mortality rates were almost halved. The greatest declines have been observed in the WHO African Region, where the burden of disease remains highest [1]. These impressive reductions have been achieved largely due to the widespread deployment of insecticide-treated nets (ITNs) and indoor residual spraying (IRS) of insecticides, which target *Anopheles* malaria vectors. However, the effectiveness of these core malaria interventions is threatened by increases in the distribution and strength (intensity) of insecticide resistance in these mosquitoes [2-4]. This is of particular concern for pyrethroids, which are currently the only insecticides used in ITNs and are also widely applied in IRS.

**Figure 1:**
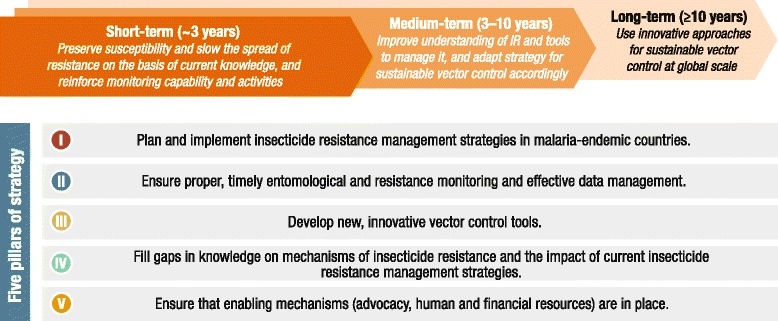
Five pillars of the Global Plan for Insecticide Resistance Management in malaria vectors. Excerpt from GPIRM [6].

In recognition of the threat of insecticide resistance, the WHO Global Malaria Programme convened an expert consultation in 2010 to inform the development of an appropriate and comprehensive response to insecticide resistance [5]. GPIRM was prepared, and released in May 2012 [6]. The Plan is a collective strategy aimed at maintaining the effectiveness of malaria vector control, and is comprised of five pillars to guide global, regional and national action in the short-, medium- and long-term (Figure 1).

**Figure 2:**
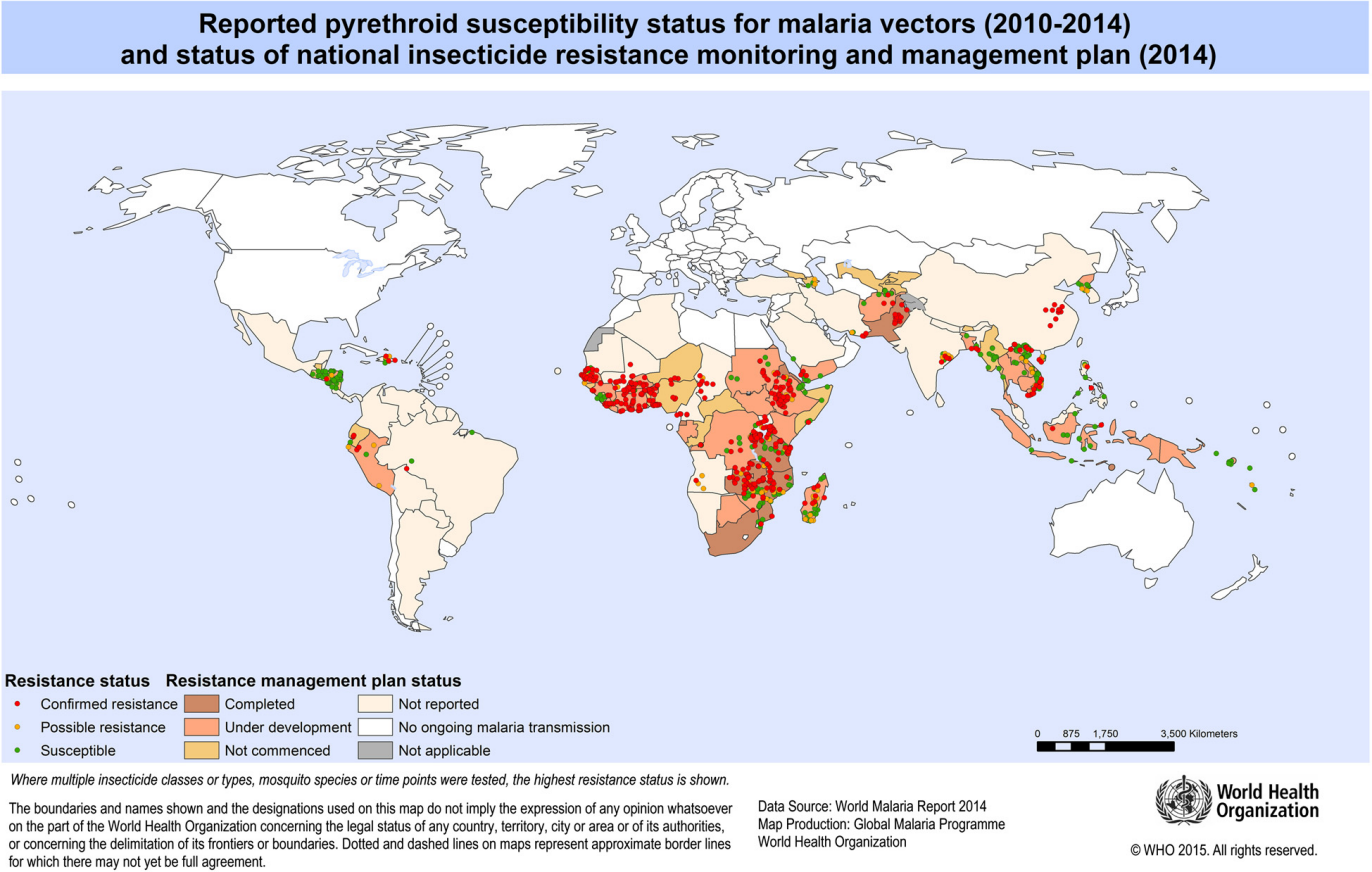
Status of pyrethroid resistance in malaria vectors and national insecticide resistance monitoring and management plans. Reported resistance status based on standard WHO susceptibility tests and CDC bottle assays using criteria of confirmed resistance (<90% mortality), possible resistance requiring confirmation (90-97% mortality) and susceptibility (≥98% mortality) with the lowest mortality displayed if multiple insecticides, vectors or time points were tested for a single locality. Status of national plans based on capacity assessment reports provided to WHO, September 2014.

Since the initial consultation and the release of GPIRM the insecticide resistance situation has worsened, particularly in the WHO African Region. Information from the recently-established WHO global insecticide resistance database indicates that since 2010, pyrethroid resistance has been detected in at least one *Anopheles* malaria vector species in 78% of countries that reported monitoring data (Figure 2); resistance to two or more insecticide classes was reported for 80% of those [1]. Stronger resistance mechanisms have been detected in *Anopheles gambiae s.s.* from West Africa, in addition to the target site mutations and metabolic-based mechanisms identified previously [7]. This has resulted in elevated levels of resistance rising up to 1,000-fold and the emergence of cross-resistance to additional insecticides [8]. There is emerging evidence that insecticide resistance is already compromising the effectiveness of malaria control efforts [3,9,10]. Meanwhile, the arsenal of WHO-recommended insecticides has remained limited to pyrethroids for ITNs, with these and three additional insecticide classes recommended for IRS. Five classes of larvicides are also recommended, though their use is not widespread.

Against this background of escalating resistance and limited vector control tools, as well as global finances that continue to fall short of estimated requirements for malaria control and elimination [1] and restricted entomological capacity [11], there has been some progress in the implementation of GPIRM. This case study reviews the progress made, the challenges experienced to date, and proposes key actions for accelerating global efforts to provide sustainable universal access to effective malaria vector control. It builds on the report issued to the Malaria Policy Advisory Committee (MPAC) in September 2014 [12] and provides additional data on insecticide resistance and entomological capacity obtained from WHO Member States in 2014.

### PILLAR I Plan and implement insecticide resistance management strategies in malaria-endemic countries

Seven countries in Africa reported having established a national insecticide resistance monitoring and management plan by September 2014: Equatorial Guinea, Eritrea, Mozambique, Rwanda, South Africa, United Republic of Tanzania, and Zambia. Plans are being developed in most other countries in sub-Saharan Africa but this process has yet to be commenced in the vast majority of countries outside of the region. The quality and viability of existing plans is also variable, with few formulated on the basis of pre-emptive action to prevent resistance emergence but most instead developed as a response to detected resistance.

To address the need for additional and better quality plans, a framework that outlines the structure and content required for a national plan was developed by WHO in 2014 and is undergoing field testing [13]. National insecticide resistance management strategies should be formulated on the basis of existing vector control interventions, status of insecticide resistance and epidemiological context [6]. In principle, good resistance management practice requires the application of multiple insecticides of different biochemical modes of action (MOA) in rotations, mosaics, mixtures, or by combining multiple interventions [6]. In practice, pyrethroid long-lasting insecticidal nets (LLINs) are the principal malaria vector control tool used in the majority of malaria-endemic settings and therefore IRS offers the main option for resistance management. Moreover, insecticides of only two biochemical MOA [14] are currently recommended by WHO for IRS in malaria vector control: sodium channel modulators (pyrethroids, DDT) and acetylcholinesterase inhibitors (carbamates, organophosphates) [15]. These insecticide formulations are also recommended for the control of other indoor-resting and -biting vectors.

Rotation of IRS insecticides by MOA on an annual basis is currently the best practice for resistance management in malaria vectors in most settings. Mosaics are often not operationally or financially feasible due to the need for multiple procurements from different sources, and variations in training and waste disposal procedures. Mixtures are not yet available for IRS due to the potential for increased cost and the difficulty of developing formulations that contain multiple active ingredients with long-lasting residual activity, positive synergistic interaction and acceptable safety profiles. Combining IRS and ITNs specifically for resistance management purposes [16] may be difficult to justify financially where there are insufficient resources to cover the at-risk population with a single and effective vector control tool for disease control purposes. Larval source management may provide an opportunity for resistance management since there is greater diversity in MOAs of larvicides and habitat modification/manipulation can reduce overall dependence on insecticides but interventions targeting larvae are appropriate in certain settings only.

There has been a shift away from the use of pyrethroids in IRS, with seven countries that used this class in 2011/2012 reporting exclusive use of non-pyrethroids in 2013 [1]. However, changing to use of a non-pyrethroid class has invariably been driven by the detection of high-level pyrethroid resistance, rather than the proactive implementation of good resistance management practice as part of a long-term national strategy. For most countries, the change to non-pyrethroid IRS was associated with a reduction in the overall proportion of the at-risk population protected by IRS as a result of the increased cost of procuring and deploying non-pyrethroid alternatives [1]. For example, while pyrethroids require two spray rounds in areas with a malaria transmission season beyond six months and are approximately USD2-3 per sachet (for coverage of an estimated 250 square meters of surface area), a new long-lasting organophosphate (pirimiphos-methyl) formulation requires one spray round per season and is approximately USD23 for equivalent coverage. Once application costs are taken into account, the cost of spraying one round of the long-lasting organophosphate formulation may be similar to that required for two rounds of a pyrethroid. However, in reality, even countries with perennial transmission often do not spray with sufficient frequency to ensure year-round effective coverage.

Global coverage rates of at-risk populations with IRS have decreased annually from 5% (185 million people protected) in 2010 to 4% (124 million) in 2013 [1]. As the use of IRS declines, so too do the opportunities for insecticide resistance management using current tools.

### PILLAR II Ensure proper, timely entomological and resistance monitoring and effective data management

GPIRM outlined the importance of routinely collecting, analysing, managing, and sharing data on insecticide resistance to support timely and informed programmatic decision-making. For 2013, 77 countries reported conducting insecticide resistance monitoring of some form, although monitoring data were provided to WHO by only 42 countries [1]. This disparity is likely due to the fact that resistance monitoring is not necessarily conducted on an annual basis and there may be delays or barriers to reporting all monitoring data to the national malaria control programme within three months of testing, as recommended in GPIRM.

When conducted, resistance monitoring seldom includes testing of all major malaria vector species with each of the four insecticide classes. Lack of continuity in data collection precludes the identification of temporal and spatial trends. Issues with the consolidation and dissemination of monitoring data at the national level are often due to reticence of those who conduct monitoring to share the data for various reasons, including a misconception that this will prevent publication in scientific journals.

It is essential that national programmes are at the centre of any monitoring initiatives, including the management and dissemination of arising data, since the impetus is for this to improve the impact of vector control. A generally low capacity in data management within national malaria control programmes is however a major limitation in many countries; in 2014, only 34 malaria-endemic countries reported the existence of a national insecticide resistance database with seven countries planning to develop a database in 2014/2015. It is important that partners supporting or conducting insecticide resistance monitoring also invest in building the necessary national capacity and structures to manage the resistance data.

A global database has been established by WHO at the request of Member States to consolidate insecticide resistance data reported by countries and partners supporting monitoring along with data extracted from scientific publications. By the end of 2014, the global database contained information from 2,019 sites in 71 countries for 110 vector species from both WHO susceptibility assays and USA Centers for Disease Control and Prevention (CDC) bottle bioassays. Limited data were included on resistance mechanisms, although the published literature contains extensive information particularly for countries such as Burkina Faso, Benin and Kenya [17]. Consolidation of available resistance mechanism data will be undertaken in 2015. Plans are in progress to develop interactive maps to display selected aggregate national data and geo-referenced sub-national data, due to the complexity of interpreting information from multiple years, species, insecticides, or mechanisms. These information management tools will facilitate data sharing and timely availability to guide national and global malaria policy.

An updated version of the test procedures for insecticide resistance monitoring in malaria vector mosquitoes was released in April 2013 [18]. This has served to improve standardization of testing and reporting. However, current bioassay tests which measure mortality in response to a fixed concentration of a given insecticide over a set time period are liable to variation depending on test conditions (e.g., temperature, humidity, age of test mosquitoes), and whilst they may give an indication of resistance frequency according to set criteria, they are not sufficient for measuring resistance strength or predicting impact on intervention efficacy. Additional resistance testing methods are clearly needed; their development and uptake must be informed by sufficient cross-laboratory and field validation. For example, evidence on the use of the CDC bottle bioassay to measure resistance strength will be reviewed by the WHO Vector Control Technical Expert Group (VCTEG) to determine its utility and practicality for routine insecticide resistance monitoring by control programmes.

Development of technical competency through training is also key to supporting sound insecticide resistance monitoring and management. A number of regional and national training courses focussed on insecticide resistance have been conducted since the release of GPIRM. Numerous partners have been commendably involved in coordinating training workshops on insecticide resistance monitoring and have assisted with provision of the necessary equipment to the countries in which they operate. These have focused on imparting knowledge and skills to national technicians on the collection of data, rather than the skills to correctly analyse, interpret and manage the data to better inform control. The critical need remains in many countries for resources and human capacity to collect, manage and share entomological data as well as use the data appropriately [11] to guide the management of insecticide resistance. Resources are also required within WHO to support countries in coordinating the implementation of technical recommendations outlined in GPIRM and other relevant vector control policies.

### Pillar III Develop new, innovative vector control tools

Whilst current insecticide resistance management efforts focus on judicious use of existing interventions, the development and adoption of LLIN and IRS formulations with new MOA is essential for resistance management in the medium- to long-term. Biological and environmental appropriateness are also key considerations, and development must be guided by defined target product profiles (TPP), with validation including social compliance (acceptability) and assessment of scalability and cost [19]. The pipeline of new insecticide-based vector control products has dramatically improved in the past ten years, mainly due to the Innovative Vector Control Consortium (IVCC) product development partnership (Table 1). In the longer term, new interventions are also needed to address residual malaria transmission whilst providing effective options for insecticide resistance management.

**Table 1:** **New insecticidal products for malaria vector control**

**Intervention**	**Status**
**Indoor residual sprays**	Two new long-lasting formulations of existing IRS insecticides but with increased longevity beyond the benchmark of 2–4 months, to 6–12 months have already reached the market. Other formulations of repurposed agro-chemicals are under development, but are at best 12–24 months from becoming available for deployment. IVCC has established a portfolio of novel active ingredient candidates that should deliver new public health insecticides by 2022.
**Long-lasting insecticidal nets**	New formulations are in preparation, with the first generation of these containing a pyrethroid plus a synergist or growth regulator. An important step will be to examine potential additional benefits against pyrethroid-resistant *Anopheles*. A second generation of non-pyrethroid multi-insecticide nets is in early stage development but it is likely to be several years before availability for wide-scale deployment.
**Spatial repellents**	Currently there are insufficient data to assess whether spatial repellents could play a substantive role in malaria disease prevention. A multi-country field trial of the effectiveness of repellents is under way which should establish whether repellents work against most or just a small sub-set of mosquito vectors, but this study is unlikely to alone provide sufficient evidence to recommend wide-scale usage of repellents as part of national control programmes. Continued commitment from industry and research groups will be required to identify and validate any promising new candidates.

Information provided by the IVCC.

For novel or improved interventions to reach the market and become available for implementation in the predicted timeframe, the global and national regulatory framework, including efficacy and safety assessments, will need to be adapted since these are often appropriate for existing vector control paradigms only. The successful deployment of new tools and strategies will require multisectoral coordination across numerous stakeholder groups, led by national malaria control programmes and including national registration bodies, environmental ministries, researchers, and vector control commodity suppliers. Continuity in the core group will ensure knowledge and experience retention, though supplementary support from external experts may be sought as required. Alongside innovation in product development and harmonization of policy and and regulatory processes funding mechanisms and supply chains must be optimized to achieve cost-effective and sustainable vector control.

To aid development and ensure appropriate technical recommendation for new vector control paradigms, the WHO Vector Control Advisory Group (VCAG) was constituted in 2012. The group is jointly managed by the WHO Global Malaria Programme and the Department of Control of Neglected Tropical Diseases, and assesses the potential public health benefits of new paradigms, tools and technologies for vector control. Following an initial recommendation on the new paradigm, an over-arching TPP can be established and the WHO Pesticide Evaluation Scheme may then proceed with the validation of individual product safety and efficacy using product specifications derived from this TPP. The outcome of the VCAG process will be to shorten the time from development to deployment of newly validated vector control tools to protect populations from malaria and other vector-borne diseases. To date, VCAG has established its working procedures and has reviewed 18 dossiers from innovators of potential tools and technologies of public health importance; a list of paradigms currently under consideration can be found on the website [20]. VCAG is currently developing guidelines on the minimum data required for evaluating products of new paradigms including LLINs with a claim of improved efficacy against pyrethroid-resistant malaria vectors, and the VCTEG is developing guidance defining operational conditions for their deployment.

### Pillar IV Fill gaps in knowledge on mechanisms of insecticide resistance and the impact of current insecticide resistance management approaches

GPIRM sets out priorities for research in the short-, medium- and long-term, although it emphasized that the lack of full information and evidence in some key areas need not preclude pre-emptive action to address insecticide resistance. Since the release of GPIRM, progress has been made in some but not all priority areas.

Evidence on sub-regional and regional trends in the spread of resistance in locally important vector species is being compiled through WHO global and regional databases, and will be reviewed periodically. There has been significant investment by various institutes in characterizing and monitoring metabolic resistance mechanisms in recent years; consequently, the time required to identify the underlying causes of resistance has been reduced from six to 12 months to a matter of weeks, although the process still requires technical and infrastructural capacity that exceeds what is currently available in most malaria-endemic countries. The establishment or strengthening of country reference centres or regional centres of excellence, along with mechanisms to provide sustainable resources to support scientists in malaria-endemic countries, are needed to avail the necessary evidence-base for sustainable vector control.

Assessing the impact of insecticide resistance on the effectiveness of interventions is an essential but difficult task. A number of studies on ITNs claiming to have evaluated this have yielded differing results. Evaluations of the impact of IRS in areas with LLINs and pyrethroid-resistant *Anopheles* have also provided different outcomes [16]. The Roll Back Malaria Partnership (RBM) Vector Control Working Group commissioned a systematic review of evidence on the impact of pyrethroid resistance on ITN efficacy and malaria transmission [21]. It found that poor standardization of methodologies, inadequate controls and poor or no characterization of underlying resistance mechanisms in most studies precluded a definitive conclusion on the impact of resistance on entomological outcomes or disease transmission. To address these limitations, a major multi-country study was undertaken primarily to ascertain the impact of insecticide resistance on the effectiveness of LLINs and IRS. One of the main challenges has been the inability to randomly allocate insecticide resistance clusters within trials, necessitating prospective observational studies of large scale to ensure sufficient statistical power [6,22]. The project coordinated by WHO is being implemented in Benin, Cameroon, India, Kenya, and Sudan and will be completed in 2016. Interim results suggest that there are variations in the impact of insecticide resistance on the effectiveness of LLINs versus IRS, and between different settings [22].

There remains a paucity of evidence on the utility of conventional resistance management strategies (e.g., insecticide rotations, mosaics, mixtures, and combinations) in restoring the susceptibility of malaria vectors. There is also a need for well-designed assessments of the operational impact of combinations of insecticidal and non-insecticidal interventions, including larval source management approaches. Outcomes are likely to be dependent on the levels of malaria parasite transmission, the behaviour and the resistance profile (including frequency, intensity and type of mechanisms) of local mosquitoes, and coverage of interventions. Evaluations of candidate approaches should focus on validated cost-effective interventions that can be implemented at scale within the logistical and financial constraints of the national malaria control programmes. Consideration should also be given to economic constraints or opportunities, such as decreasing commodity and implementation costs that may result during scale-up.

### Pillar V Ensure that enabling mechanisms (advocacy, human and financial resources) are in place

High-level representatives from all key constituencies of the global malaria community participated in the launch of GPIRM in May 2012. The executive summary of GPIRM was made available in English, French and Spanish in hard copy and on the WHO website, and was circulated widely through various avenues including RBM working groups and sub-regional networks. The World Malaria Report 2014 [1] includes a section that summarizes the status of insecticide resistance on the basis of information from the newly established WHO global insecticide resistance database.

These advocacy initiatives have catalysed open engagement and communication on the extent and implications of insecticide resistance, yet the human and financial resources committed to tackling this problem are inadequate. In parallel to the development and dissemination of GPIRM, efforts to mobilize financial resources to support its implementation were undertaken by various groups, including WHO, RBM and CDC. These included approaching traditional donors as well as exploring innovative ways to engage non-traditional donors for independent management of funds from sources including the private sector. While this resulted in investments for some activities such as resistance monitoring and global databases, these fell far short of the necessary finances required to support GPIRM implementation. This is in stark contrast to the efforts and resources that have been committed to curtailing the spread of malaria parasite resistance [23].

One of the major limitations to widespread support for GPIRM has been the lack of availability of non-pyrethroid LLINs and options for IRS restricted to only two MOA. Furthermore, access to IRS formulations of acetylcholinesterase inhibitors (organophosphates and carbamates) has largely been limited by their high cost relative to pyrethroids; their use has often necessitated a reduction in IRS coverage due to a lack of financial resources to compensate for the cost increase. This barrier to access has been exacerbated by the lack of evidence on comparative cost-effectiveness of IRS formulations with different residual efficacy, and limited capacity at the country level to use such evidence for local decision-making.

Attempts by WHO to engage industry partners on potential price concessions for existing or new IRS products have had limited success. The US President’s Malaria Initiative also attempted to discuss pricing with industry, and has advocated for insecticide manufacturers to look into price elasticity models, i.e., if price goes down, quantity purchased goes up to reach an equilibrium point. This has not been successful in part because a high commodity price and a single supplier for the only long-lasting, non-pyrethroid IRS formulation has led to a small, chaotic marketplace and low uptake, resulting in a lack of reliable, long-term demand forecasting. Using their experience with LLIN procurement, the Global Fund to Fight AIDS, Tuberculosis and Malaria is considering engaging with industry on a new procurement strategy for IRS in 2015. It should be noted that the basic cost of manufacture of the non-pyrethroid alternatives, whatever the volume, will nevertheless be significantly higher than that of pyrethroids. The factors limiting programme access to non-pyrethroids, such as reductions in costs for the overall management and implementation of quality IRS, must be addressed in order to achieve and sustain universal access to effective malaria vector control.

## Conclusions and the way forward

For most malaria-endemic countries, and particularly those in Africa south of the Sahara, pyrethroid resistance in malaria vectors is worsening. There are also increasing reports of resistance to organophosphates, carbamates and DDT. Pre-emptive action against insecticide resistance as emphasized in GPIRM is still the goal, but immediate measures are needed to address pyrethroid resistance. However, the options for LLINs and IRS remain limited, which further challenges the goal of preserving the effectiveness of malaria vector control and providing universal access of at-risk populations to malaria prevention. Some progress has been made in implementing GPIRM. There has been enhancement in capacity and resources for insecticide resistance monitoring [1], development of new IRS formulations with extended efficacy and the establishment of global and regional insecticide resistance databases. However, whilst some countries have switched from using pyrethroids in IRS, most have yet to establish and implement national insecticide resistance monitoring and management plans that incorporate ongoing rotation of insecticides with different MOA. Key limitations to the uptake of GPIRM have been the lack of vector control tools with new MOA, and major financial, human and infrastructural resource deficiencies.

Until non-pyrethroid, multi-insecticide LLINs are available, insecticide resistance will, to a great extent, rely on the targeted use of rotational IRS with different MOA. However, a decline in the global at-risk population protected with IRS restricts options for resistance management. With the high cost of current pyrethroid alternatives being one of the main barriers to implementing GPIRM technical recommendations, it is vital that options for improving affordability are urgently pursued. Better global forecasting of insecticide requirements, pooled procurement and long-term agreements and tax-free incentives have been successfully applied to the LLIN market. These approaches may be feasible to enhance the confidence of IRS chemical manufacturers, help stabilize the market and eventually lead to price reductions. Together, these actions may support the maintenance and/or scale-up of IRS for insecticide resistance management purposes, which must be conducted in parallel with enhanced entomological surveillance coupled with efficient data management to inform programmatic decisions. Moreover, tracking the status of insecticide resistance and progress in GPIRM implementation will require the identification and regular measurement of both national and global key indicators.

The majority of countries currently implementing IRS depend heavily on external donor support, especially in Africa. Countries and partners are therefore urged to develop and implement national insecticide resistance monitoring and management plans that include contingencies for ongoing use of more expensive alternative IRS insecticides as part of national strategic plans. Resource mobilization should also be pursued from non-traditional donors to ensure the full cost of deploying non-pyrethroid IRS is covered, and can be justified on the basis that programmatic cost increases will be inevitable in the face of resistance. There is a need for enhanced investments by endemic countries coupled with strategic plans for transitioning to full financing and management of vector control activities wherever feasible.

In parallel to these efforts, additional investments should be made to build country capacity to monitor insecticide resistance, including quantifying resistance intensity and assessing its operational impact. Often the need for and actual role of public health entomologists supporting malaria control programmes is not clear to those at higher management levels. The skills set required for senior personnel coordinating entomological surveillance as well as vector control implementation, monitoring and evaluation (which may require different staff and expertise) should be clearly defined on the basis of programme needs.

Country reference centres run in collaboration with the national malaria control programme should be established, potentially by upgrading existing institutions with the necessary facilities. The complexity of characterizing the underlying resistance mechanisms means that establishing capacity for these assessments, which will require additional investments, will not be practical in all malaria-endemic countries. Country, regional or global centres that can rapidly assess mechanisms and feedback results in a timely manner should be established to work alongside national programmes to ensure optimal uptake and use of information. This will help in building capacity of scientists from developing countries, particularly those working in national malaria control programmes. A mechanism is also needed to ensure that trained scientists are empowered to utilize skills in their own countries that have been acquired elsewhere. Specialized re-entry grants, such as those issued by the WHO Special Programme for Research and Training in Tropical Diseases, should be implemented to address this problem.

WHO must support these initiatives by building awareness and consensus around the extent of the problem of insecticide resistance - similar to those efforts with artemisinin resistance - and should explore ways to ensure that alternative products for managing insecticide resistance are affordable. With the current knowledge and experience, it would be irresponsible for the global community to wait until malaria programmes report increases in malaria cases due to insecticide resistance before there is a significant response. Moreover, to continue to witness declines in IRS coverage due to a lack of incorporating non-pyrethroids is unacceptable. Global inaction deprives affected communities of their basic right of universal access to effective protection against malaria. A global response plan for insecticide resistance in malaria vectors is under development by WHO. Using GPIRM as the technical basis, this will clearly outline the actions required by national malaria control programmes and their partners and the indicators to track progress in addressing the challenge of insecticide resistance.

## Abbreviations

CDC: Centers for Disease Control and Prevention
DDT: Dichlorodiphenyltrichloroethane
IRS: Indoor residual spraying
ITN: Insecticide-treated net
IVCC: Innovative vector control consortium
GPIRM: Global Plan for Insecticide Resistance Management in malaria vectors
LLIN: Long-lasting insecticidal nets
MPAC: Malaria Policy Advisory Committee
MOA: Mode of action
RBM: Roll Back Malaria partnership
TPP: Target product profile
VCTEG: Vector Control Technical Expert Group
VCAG: Vector Control Advisory Group
WHO: World Health Organization
